# Photocatalytic degradation kinetics of Orange G dye over ZnO and Ag/ZnO thin film catalysts

**DOI:** 10.1038/s41598-019-54142-w

**Published:** 2019-11-26

**Authors:** Derya Tekin, Taner Tekin, Hakan Kiziltas

**Affiliations:** 1 Department of Metallurgical and Materials Engineering, Faculty of Engineering, Ataturk University, Erzurum, Turkey; 2 Department of Chemical Engineering, Faculty of Engineering, Ataturk University, Erzurum, Turkey

**Keywords:** Pollution remediation, Chemical engineering

## Abstract

The degradation of water pollutants with photocatalysts is one of the most studied subjects in the past 20 years. Although considerable studies have been completed in this field, kinetic model studies are still a major inadequacy. In this study, ZnO and Ag/ZnO thin film photocatalysts were synthesized and SEM-EDS, XRD and chronoamperometric measurements were used the characterization of photocatalysts. The network kinetic model was applied the photocatalytic degradation of Orange G using ZnO and Ag/ZnO thin film photocatalysts. The photocatalytic degradation of Orange G was investigated under the different reaction medium (initial dye concentrations, temperature, light intensity). It was found that the network kinetic model is the most appropriate model for the degradation of Orange G dye on the ZnO and Ag/ZnO thin film photocatalysts. The calculated adsorption equilibrium (K_B_) constant and activation energy of ZnO thin film photocatalyst are 0.0191 and 21.76 kj/mol, respectively. Additionally, the calculated values for Ag/ZnO thin film photocatalyst are 0.035 and 18.32 kj/mol. The general rate equations were determined for each photocatalysts.

## Introduction

As a result of the colorful products increasing the trade of people, the applications of colorful products in different areas are rapidly increasing. In particular, organic dyes released from cosmetics, food, textiles, pharmaceuticals industries form a large proportion of the contaminants in the wastewater^1^. The release of organic dyes, which has high stability, to the environment causes ecological problems and threatens the living life in the water^2^.

The photocatalytic systems are very effective processes for the treatment of wastewater in the presence of semiconductor photocatalysts^3^. Although it is known that the most effective photocatalyst is titanium dioxide (TiO_2_) among semiconductor photocatalysts, some studies have shown that zinc oxide (ZnO) is more effective than TiO_2_ in photocatalytic degradation of some dyes^4,5^. However, the properties, such as high recombination rate of the electron hole pairs and low interfacial charge carrier transfer rate, limit the using ZnO in the photocatalytic applications^6^. Various methods, such as doping of metals and non-metals^7^, combining with different semiconductors^8^, are used to overcome these negative features. The metal doping of ZnO is most commonly used method to improve its photocatalytic efficiency, because metal doping may change its electrical, optical and magnetic properties^9^.

Although some noble metals, such as Pd, Pt, Au, were used to improve the photocatalytic activities of photocatalysts, their high cost limits the large-scale applications^10^. The use of silver, also a noble metal, as a doped material is sufficient to eliminate this disadvantage.

In this study, the solutions of ZnO and Ag/ZnO photocatalysts were synthesized by sol-gel method, and ZnO and Ag/ZnO thin film photocatalysts were coated on the quartz tubes by dip-coating method. The characterizations of prepared thin-film photocatalysts were performed using SEM-EDS, XRD, chronoamperometric measurements. For the kinetic studies, the degradation over the produced photocatalysts of Orange G dyestuff was investigated.

## Experimental

Zinc acetate dihydrate (ZnAc, purity above 98%), silver nitrate (purity above 99%), diethanolamine (purity above 98%) and 2-Propanol (99.5% purity) were used for the synthesis of ZnO and Ag/ZnO photocatalysts. Acetone (purity above 97%), ethanol (99.9% purity) and methanol (99.8% purity) were used for cleaning of the quartz tubes. Orange G was used for photocatalytic experiments. All chemical materials were purchased from Sigma-Aldrich.

### Preparation of ZnO solution

The zinc acetate dihydrate salt (52.5 gr) was dissolved in 2-propanol (600 ml) under 1000 rpm stirring speed for 2 hours at 65 °C. Diethanolamine (39 ml) was added dropwise to the stirring solution and stirred at room temperature for 2 hours.

### Preparation of Ag/ZnO solution

The zinc acetate dihydrate salt (52.5 gr) was dissolved in 2-propanol (600 ml) under 1000 rpm stirring speed for 2 hours at 65 °C. Diethanolamine (39 ml) was added dropwise to the stirring solution and stirred at room temperature for 1 hour. Then, silver nitrate (0.325 gr) was added the stirred solution, and the new solution was stirred for 1 hour.

### Coting procedure

The quartz tubes were ultrasonically cleaned in acetone, methanol, ethanol, and deionized water, respectively. Then, the dried tubes were dipped into the pre-prepared photocatalyst solution at a rate of 10 cm/min and angle of four degrees, and withdrawn at the same rate. The coating cycle was repeated ten times. Owing to the fact that the maximum efficiency of ZnO thin-film photocatalyst was achieved as a result of the calcination process at 450 °C in the literature, the calcination temperature of the ZnO thin-film photocatalyst was chosen to be 450 °C^11^. Due to the fact that the optimum photocatalytic results of Ag-ZnO thin-film photocatalyst was obtained as a result of the calcination process at 400 °C in the literature, the calcination temperature of the Ag-ZnO thin-film photocatalyst was chosen to be 400 °C^12^. The coating area of quartz tubes is approximately 30 cm^2^.

### Photocatalytic measurements

Photocatalytic measurements of the produced thin-film photocatalysts were performed using Orange G dyestuff. In the experimental system, a batch reactor with light isolation was used. The reactor medium was kept constant temperature with a water circulator. An air pump was used to provide the saturated O_2_ concentration in the reactor. The effect of initial dye concentrations (20, 30, 35 and 40 ppm), temperatures (20, 30, 40 and 50 °C) and light intensities (44, 88 and 132 W/m^2^) were investigated in the experiment for each thin film photocatalysts. The Orange G dye concentrations at different times were measured by using UV-vis spectrophotometer at 474 nm (Evolution 500, Thermo). The experiments were repeated in the dark medium to determine whether the dye removal was due to adsorption. It is observed that the dye decomposition was only due to photocatalytic degradation because the significant changes in dye concentration were not occurred in the dark medium experiments. The stability of produced thin film photocatalysts was determined with repetitive experiments.

### Characterization

The thin film photocatalysts were characterized to determine the surface morphology by using Scanning Electron Microscope (SEM) (Inspect S50, FEI, Czech Republic), Energy-Dispersive X-ray Spectroscopy (EDS) (Inspect S50, FEI, Czech Republic). The X-ray diffraction (XRD) (D/Max-2200, Rigaku, Japan) analysis was used to determine the crystal structures of each thin film photocatalysts. The potentiostat system (VersaSTAT3, Amedek, Ireland) was used with the linear sweep voltammetry experiments.

## Result and Discussion

**Figure 1:**
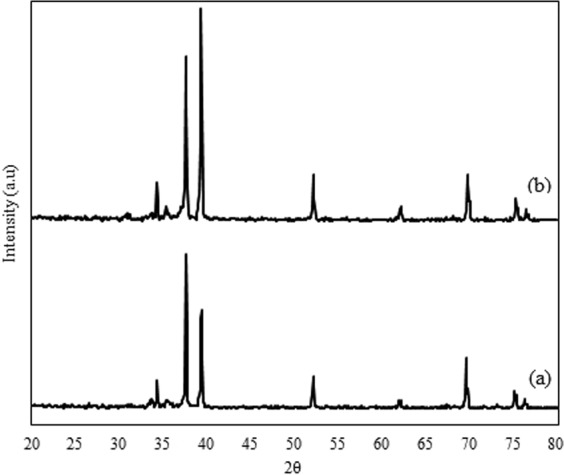
XRD results of ZnO (a) and Ag/ZnO Photocatalysts.

Figure 1 shows the XRD results of the ZnO and Ag/ZnO thin film photocatalysts. As can be seen from Fig. 1a, all detectable peaks can be ascribed for the ZnO wurtzite structure (JCPDS 36-1451. As shown in Fig. 1b, the increasing diffraction peak shown at 38° could be indexed to the face centered cubic structure of metallic Ag for Ag/ZnO thin film photocatalyst (JCPDS 04-0783). In addition, the substitution of Ag atoms in ZnO hexagonal lattices caused a slight shift in the diffraction peak^13^.

**Figure 2:**
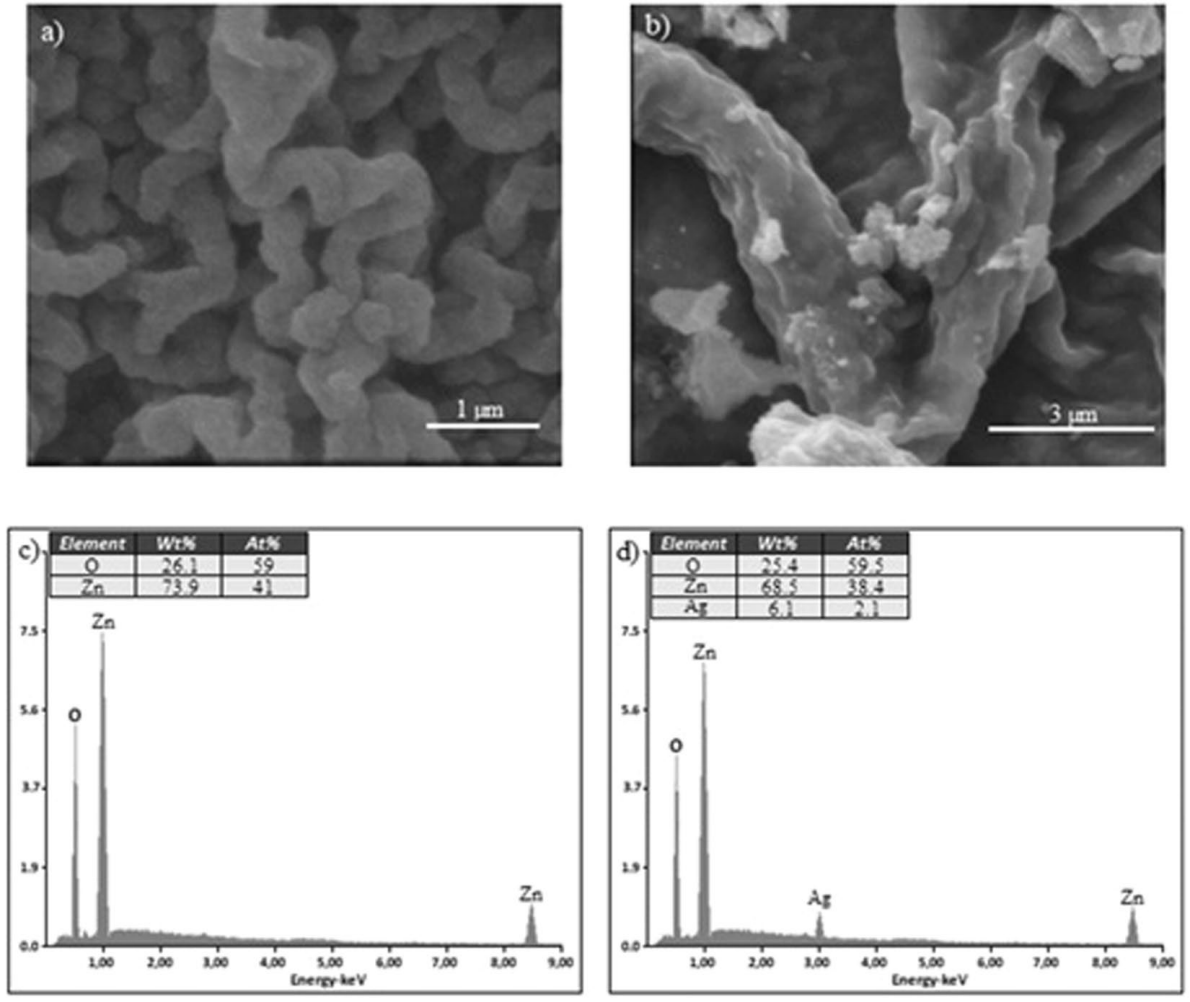
SEM images and EDS analysis of ZnO (**a**–**c**) and Ag/ZnO (**b**–**d**) Photocatalysts.

Figure 2 shows SEM images and EDS analysis of ZnO and Ag/ZnO thin film photocatalysts. ZnO and Ag/ZnO thin film photocatalysts have a wrinkled structure shown in Fig. 2a,b. The formation of this structure is formed due to the differences in the thermal expansion coefficients between the film materials and the substrate under the calcination process^14^. The EDS analyzes shown in Fig. 2c,d indicate presence of Zn and O for ZnO thin film photocatalyst and presence of Zn, O and Ag for Ag/ZnO thin film photocatalyst. The interaction between Ag and ZnO can be proven with the shift of absorption spectra^15^.

**Figure 3:**
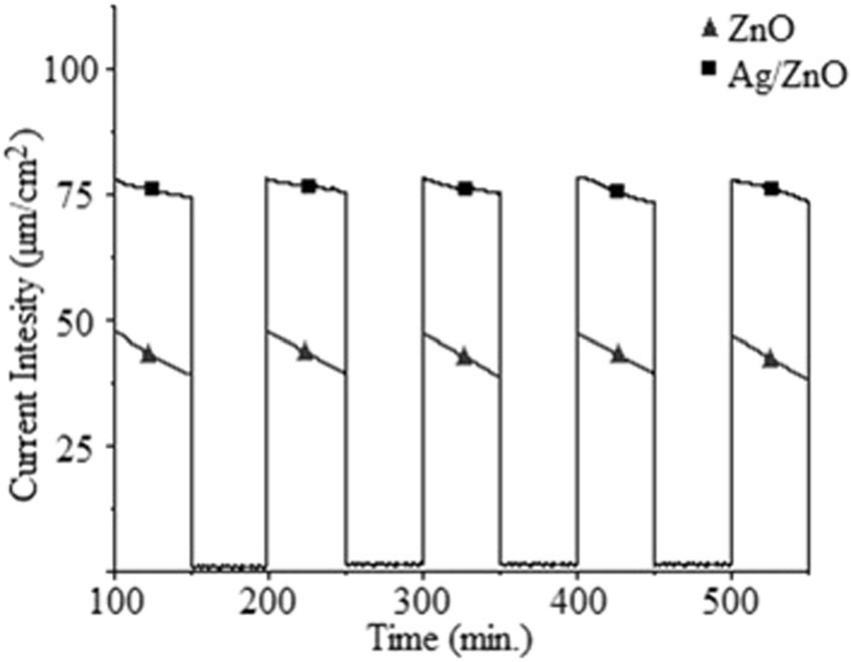
The linear sweep voltammetry results of ZnO, Ag/ZnO thin film photocatalyst.

The linear sweep voltammetry experiments were achieved under the UV-light irradiation and dark medium as shown Fig. 3. The formation of current was exclusively generated under UV light irradiation because the current intensity was approximately zero in the dark medium. The current densities of the ZnO and Ag/ZnO thin film photocatalysts are approximately 45 and 75 µA/cm^2^, respectively. The results show that the Ag/ZnO thin film photocatalyst shows higher current density than ZnO thin film photocatalyst at the same condition.

In the general photocatalytic degradation experiment, the hydroxyl radicals were adsorbed on the active sites of photocatalysts, attack the organic molecules and degrade them. The elementary reaction mechanism is summarized in the following:

1 $${\rm{Z}}{\rm{n}}{\rm{O}}+{\rm{h}}\nu \to {\rm{Z}}{\rm{n}}{\rm{O}}({{\rm{e}}}^{-}+{{\rm{h}}}^{+})$$

2 $${\rm{ZnO}}({{\rm{h}}}^{+})+{{\rm{H}}}_{2}{\rm{O}}\to {\rm{ZnO}}({{\rm{OH}}}^{\cdot })+{{\rm{H}}}^{+}$$

3 $${\rm{ZnO}}({{\rm{e}}}^{-})+{{\rm{O}}}_{2}\to {\rm{ZnO}}({{\rm{O}}}_{2}^{-\cdot })$$

4 $${\rm{ZnO}}({{\rm{OH}}}^{\cdot })+{{\rm{C}}}_{{\rm{dye}}}\to {\rm{Products}}+{\rm{ZnO}}$$

5 $${\rm{ZnO}}({{{\rm{O}}}_{2}}^{-\cdot })+{{\rm{C}}}_{{\rm{dye}}}\to {\rm{Products}}+{\rm{ZnO}}$$

It has been shown that the pseudo-first kinetic model frequently used for the degradation kinetics of dyestuff is not suited to identify the effect of the experiment parameters. Therefore, the new kinetic model called the network model has been recommended^16,17^, is given below:

6 $$-\frac{d{C}_{dye}}{dt}=\frac{{k}_{a}{C}_{dye}}{(1+{k}_{b}{C}_{dye})}$$

where, k_a_ and k_b_ are model constants. The Eq. 6 was integrated with the initial condition (C_dye_ = C_0_ at t = 0) and was rearranged.

7 $$\frac{t}{({C}_{0}-{C}_{dye})}=\frac{{k}_{b}}{{k}_{a}}+\frac{1}{{k}_{a}}\frac{\mathrm{ln}({C}_{0}/{C}_{dye})}{({C}_{0}-{C}_{dye})}$$

**Figure 4:**
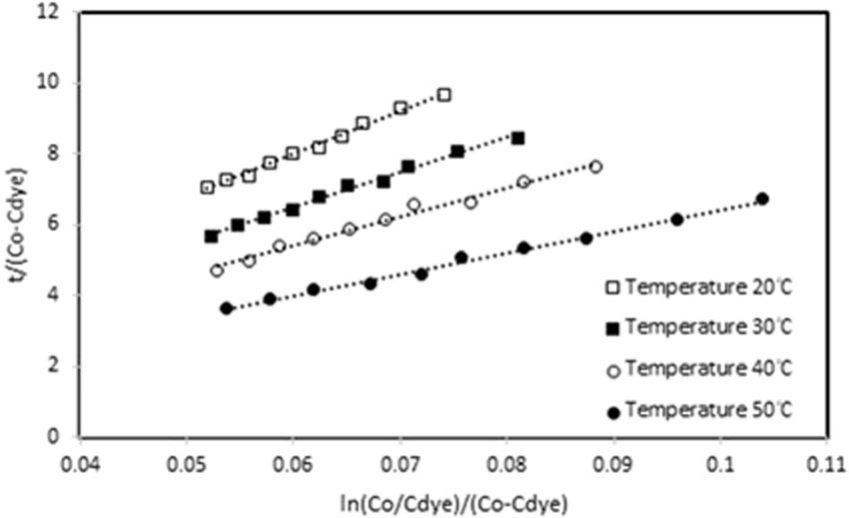
The network kinetic graph of ZnO thin film photocatalyst for temperatures.

**Figure 5:**
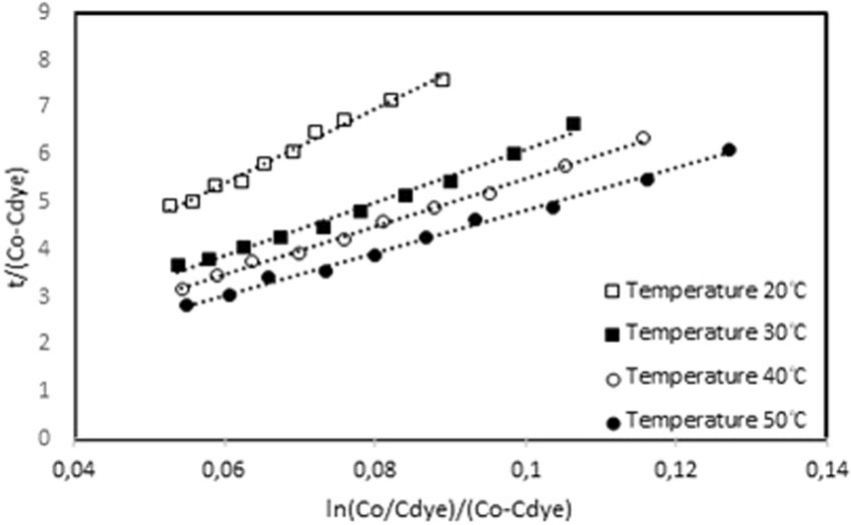
The network kinetic graph of Ag/ZnO thin film photocatalyst for temperatures.

The different experimental parameters of initial dye concentration, temperature and UV-light intensity were used to plot the graphs of t/(C_0_-C_dye_) values versus ln(C_0_/C_dye_)/(C_0_/C_dye_) values. The graphs for different temperatures were shown in Figs. 4, 5 for ZnO and Ag/ZnO thin film photocatalysts. The intercept and slope values of the graphs were used to calculate k_a_ and k_b_ constants, and given Table 1. It is obtained from the linearity of the graphs that the network kinetic is compatible for the photocatalytic decomposition kinetics of Orange G dye on ZnO and Ag/ZnO thin film photocatalysts.

**Table 1:** The k_a_ and k_b_ model constants for the degradation experiments using ZnO and Ag/ZnO thin-film photocatalyst.

Initial Dye Conc. (mg/L)	ZnO Thin-Film Photocatalyst		Ag/ZnO Thin-Film Photocatalyst	
	k_a_ Model Const. (min^−1^)	k_b_ Model Const. (L/mg)	k_a_ Model Const. (min^−1^)	k_b_ Model Const. (L/mg)
20	0.0083	0.0063	0.0129	0.00971
30	0.0076	0.0063	0.0108	0.00994
35	0.0070	0.0064	0.0097	0.00962
40	0.0065	0.0062	0.0092	0.00991
**Temperature (πC)**				
20	0.0083	0.0063	0.0129	0.00971
30	0.0103	0.0065	0.0180	0.00972
40	0.0123	0.0064	0.0200	0.00924
50	0.0168	0.0065	0.0253	0.00966
**Light Intensity (W/m**^**2**^**)**				
44	0.0083	0.0063	0.0129	0.00971
88	0.0118	0.0065	0.0195	0.00975
132	0.0181	0.0064	0.0284	0.00980

Although the k_b_ model constant values remain constant, the values of k_a_ model constant are varied by different experimental parameters, as seen in Table 1. In order to examine the changing in k_a_ model constant, the following Eq. 8 was proposed^17–19^.

8 $${k}_{a}=\frac{{k}_{o}\exp (\,-\,Ea/RT){I}_{a}}{1+{K}_{B}{C}_{Do}}$$

where, k_0_ is a temperature constant, I_a_ is the light constant, K_B_ is the adsorption equilibrium constant, and C_Do_ is the initial dye concentration.

The Eq. 9 shows the reaction rate constant (k_a_), which is derived by the Langmiur adsorption model. While the effect of initial dye concentration was studying, all other reaction parameters were constant.

9 $${k}_{a}\approx (\frac{{k}_{1}}{1+{K}_{B}{C}_{Do}})$$

Where, k_1_ is a constant depending on the light intensity. The following Eq. 10 is obtained by linearizing the Eq. 9.

10 $$\frac{1}{{k}_{a}}=\frac{1}{{k}_{1}}+\frac{{K}_{B}}{{{\rm{k}}}_{1}}{C}_{Do}$$

**Figure 6:**
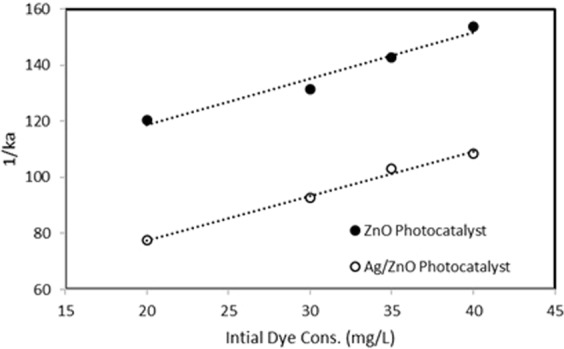
The graph of calculated 1/k_a_ values versus the initial dye concentrations.

The values of reaction rate constant (k_a_) were calculated for different initial dye concentrations (20, 30, 35, 40 ppm). As shown Fig. 6, the calculated 1/k_a_ values versus the initial dye concentrations (C_Do_) were plotted. The intercept and slope values of the graphs were used to calculate the adsorption equilibrium constant. The calculated K_B_ values are 0.0194 and 0.035 for ZnO and Ag/ZnO thin-film photocatalysts, respectively.

The Arrhenius equation given by Eq. 11 was used to investigate the temperature effect on the kinetic model and calculate the activation energy for degradation of Orange G on the ZnO and Ag/ZnO photocatalysts.

11 $${k}_{a}\approx {k}_{2}\exp (\,-\,Ea/RT)$$

**Figure 7:**
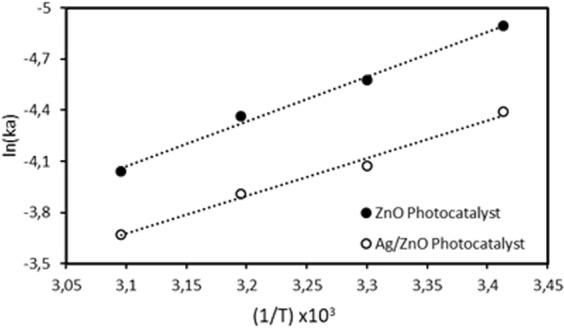
The graph of calculated ln (k_a_) values versus 1/T.

The natural logarithm of Eq. 11 was used, and the values of ln (k_a_) versus values of 1/T were plotted as shown in Fig. 7. The slopes of plotted graphs were used to compute the activation energies. The calculated activation energies (E_a_) for ZnO and Ag/ZnO thin film photocatalysts are 21.76 and 18.32 kJ/mol, respectively.

**Figure 8:**
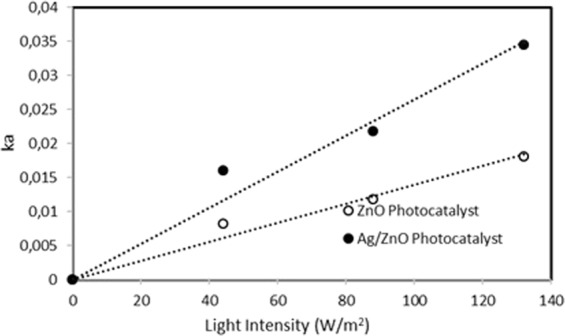
The graph of calculated k_a_ values versus light intensity.

The effect of light intensity (I_a_) was investigated by decomposition of Orange G on ZnO and Ag/ZnO photocatalysts. The value of k_a_ varies linearly with the light intensity, as shown in the Eq. 8. As shown in Fig. 8, the different light intensities (44, 88, 132 W/m^2^) were graphed against the calculated k_a_ values.

Levenberg-Marquardt method and non-linear regression analysis were used to calculate the values of k_0_. The k_0_ values were found to be 1.623 ± 0.07 for ZnO thin-film photocatalyst and 0.79 ± 0.04 for Ag/ZnO thin-film photocatalyst.

Furthermore, the prepared each photocatalysts were used in different experiments ten times under the same conditions and no change in their photocatalytic activity was determined.

## Conclusions

The ZnO and Ag/ZnO solutions were synthesis by Sol-Gel method and the dip-coating method was used to obtain ZnO and Ag/ZnO thin film photocatalysts. The prepared thin film photocatalysts were characterized by SEM - EDS, XRD and chronoamperometry. In order to eliminate the lack of literature in the kinetic study and determine the photocatalytic activity, the synthesized thin film photocatalysts were investigated on the degradation of Orange G dye. It was found that the network kinetic model is the most appropriate model for the degradation of Orange G dye on the ZnO and Ag/ZnO thin film photocatalysts. The general rate equations were determined for each photocatalysts and given below.

- The general rate equation for the degradation of Orange G dye on ZnO thin film photocatalyst:
  $$-\frac{d{C}_{dye}}{dt}=\frac{1.623\,{I}_{a}\,{(e}^{-2618/{\rm{T}}})}{(1+0.0194\,{C}_{0})}\frac{{C}_{dye}}{(1+0.0063\,{C}_{dye})}$$
- The general rate equation for the degradation of Orange G dye on Ag/ZnO thin film photocatalyst:

$$-\frac{d{C}_{dye}}{dt}=\frac{0.79\,{I}_{a}\,{(e}^{-2204/{\rm{T}}})}{(1+0.035\,{C}_{0})}\frac{{C}_{dye}}{(1+0.0097\,{C}_{dye})}$$

were obtained.
